# Associations between Antibiotics for Non-tuberculous Mycobacterial Infection and Incident Sjögren’s Syndrome: A Nationwide, Population-based Case-control Study

**DOI:** 10.1038/s41598-018-34495-4

**Published:** 2018-10-30

**Authors:** Wen-Cheng Chao, Ching-Heng Lin, Yi-Ming Chen, Chiann-Yi Hsu, Jun-Peng Chen, Hsin-Hua Chen

**Affiliations:** 1 Department of Medical Research, Taichung Veterans General Hospital, Taichung, Taiwan; 2 Division of Chest Medicine, Department of Internal Medicine, Taichung Veterans General Hospital, Taichung, Taiwan; 3 Department of Business Administration, National Changhua University of Education, Changhua, Taiwan; 4 Department of Healthcare Management, National Taipei University of Nursing and Health Sciences, Taipei, Taiwan; 5 Institute of Biomedical Science and Rong-Hsing Research Center for Translational Medicine, Chung-Hsing University, Taichung, Taiwan; 6 Division of Allergy, Immunology, and Rheumatology, Department of Internal Medicine, Taichung Veterans General Hospital, Taichung, Taiwan; 7 School of Medicine, National Yang-Ming University, Taipei, Taiwan; 8 School of Medicine, Chung-Shan Medical University, Taichung, Taiwan; 9 Institute of Public Health and Community Medicine Research Center, National Yang-Ming University, Taipei, Taiwan; 10 Department of Industrial Engineering and Enterprise Information, Tunghai University, Taichung, Taiwan

## Abstract

This study aimed to address the association between the usage of antibiotics to treat nontuberculous mycobacteria (NTM) infection and the risk of Sjögren’s syndrome (SS). We identified 5,553 patients with newly diagnosed SS between 2002 and 2013 using Taiwan’s National Health Insurance Research Database and compared them with 83,295 non-SS controls matched (1:15) for age, sex, and the year of their first SS diagnosis. An increased risk of SS was found in patients receiving new macrolides (adjusted odds ratios (aOR) 1.95, 95% confidence intervals (CI) 1.80–2.11), fluoroquinolones (aOR 1.52, 95% CI 1.41–1.64), and tetracyclines (aOR 1.69, 95% CI 1.59–1.79) compared with non-SS controls after adjusting for the Charlson comorbidity index, bronchiectasis and *Helicobacter pylori* infection. Notably, the association was consistent among each antibiotic in these three groups of antibiotics. In contrast to these three groups of antibiotics, the use of amikacin tended to have a negative association with incident SS (aOR 0.68, 95% CI 0.53–0.87). In conclusion, new macrolides, fluoroquinolones and tetracyclines were associated with a higher incidence of SS. These findings indicate the need for vigilance of SS in prescribing these antibiotics and warrant further mechanistic studies.

## Introduction

Adverse drug reactions (ADRs) are an emerging health issue and have been estimated to account for approximately 7% of admissions to hospitals^1,2^. Drug-induced autoimmunity (DIA) is a non-immunoglobulin E (IgE)-related and idiosyncratic adverse drug reaction; however, the early identification of DIA is apparently difficult, particularly in autoimmune diseases including Sjögren’s syndrome (SS), which have an insidious onset and nonspecific symptoms^3,4^. Although a wide range of drugs have been reported to potentially induce autoimmunity, only a few drugs have a clear association due to the difficulty in proving the causality in DIA and the lack of population-based research^5^. Currently, DIA has been commonly reported in drug-associated lupus based on the presence of anti-histone autoantibody, whereas DIA in diseases other than drug-associated lupus remains largely unclear^6,7^. We recently reported an association between SS and prior nontuberculous mycobacterial (NTM) infection, which was defined as a diagnosis of NTM with concurrent combinational antibiotic therapy for NTM infection^8^. However, whether the increased risk of SS was due to NTM infection or the antibiotics used to treat NTM infection is unknown. Given that combination antibiotic therapy is the fundamental strategy employed to treat NTM infections, antibiotic-associated SS might thus contribute to the development of SS. In the present study, we aimed to address the association between the use of antibiotics that are generally administered for NTM infection and the risk of newly diagnosed SS using a nationwide, population-based cohort.

## Results

### Characteristics of the study population

A total of 5,751 newly diagnosed SS patients were identified, and we further excluded those (n = 198) with a history of mycobacterial infection to avoid the potential confounding effect of NTM infection-associated incident SS that we previously identified^8^. Thus, 5,553 SS case subjects and 83,295 matched non-SS control subjects were assessed. We found that SS case subjects had a slightly higher CCI (0.5 ± 0.9 vs. 0.4 ± 1.0, p < 0.001) and were more likely to have bronchiectasis (3.6% vs. 1.1%, p < 0.001) and *H. pylori* infection (0.9% vs. 0.3%, p < 0.001) than controls (Table 1).

**Table 1:** Demographic data and clinical characteristics of the enrolled subjects.

	Control	Case	P-value
	(*n* = 83,295)	(*n* = 5,553)	
**Age, years** (mean ± SD)	55 ± 14	55 ± 14	1
**Age group**			
≤40 years	12,690 (15.2)	846 (15.2)	
40–65 years	50,175 (60.2)	3,345 (60.2)	
≥65 years	20,430 (24.5)	1,362 (24.5)	
**Gender**			1
Female	73,365 (88.1)	4,891 (88.1)	
Male	9,930 (11.9)	662 (11.9)	
**CCI**	0.4 ± 1.0	0.5 ± 0.9	<0.001
**CCI group**			<0.001
0	65,184 (78.3)	3,753 (67.6)	
≥1	18,111 (21.7)	1,800 (32.4)	
**Bronchiectasis**	922 (1.1)	197 (3.6)	<0.001
**History of** ***H. pylori*** **infection** ^a^	209 (0.3)	52 (0.9)	<0.001

^a^One year prior to the index date. Abbreviations: SS, Sjögren’s syndrome; CCI, Charlson comorbidity index; H. pylori, Helicobacter pylori; NA, non-applicable. Percentages are enclosed in parentheses after each value unless otherwise indicated.

### A high proportion of SS case subjects received new macrolides, FQNs, and tetracyclines

Patients with SS were more likely to receive new macrolides (15.8 vs. 7.1%, p < 0.001), including clarithromycin (13.7% vs. 6.4%, p < 0.001) and azithromycin (2.5% vs. 0.9%, p < 0.001), FQNs (20.0% vs. 11.8%, p < 0.001), including ofloxacin (11.2% vs. 6.8%, p < 0.001), ciprofloxacin (5.1% vs. 3.2%, p < 0.001), levofloxacin (5.1% vs. 2.6%, p < 0.001) and moxifloxacin (2.2% vs. 0.9%, p < 0.001), and tetracyclines (33.0% vs. 21.1%, p < 0.001), including doxycycline (26.6% vs. 17.0%, p < 0.001) and minocycline (11.0% vs. 6.4%, p < 0.001), than those without SS (Table 2). Given that an apparently high proportion of the enrolled subjects received tetracyclines, FQNs, and new macrolides, we further explored the major indications other than mycobacterial infections for these three groups of antibiotics. We found that acne, sinusitis, bronchitis and vaginitis/vulvovaginitis were major indications for tetracyclines; pneumonia, urinary tract infection, sinusitis and unspecified respiratory infection were major indications for the four FQNs; and *H. pylori* infection, pneumonia and bronchitis were major indications for new macrolides (Supplementary Table 1). Collectively, these data demonstrated that SS case subjects tended to have received new macrolides, FQNs and tetracyclines, and these three groups of antibiotics were frequently prescribed for pneumonia, urinary tract infection, sinusitis, and *H. pylori* infection.

**Table 2:** Administered antibiotics among enrolled subjects.

	Non-SS controls	SS cases	P-value
	(*n* = 83,295)	(*n* = 5,553)	
**New macrolide**	5,891 (7.1)	876 (15.8)	<0.001
Clarithromycin	5,304 (6.4)	763 (13.7)	<0.001
Azithromycin	714 (0.9)	141 (2.5)	<0.001
**Aminoglycoside**	1,176 (1.4)	71 (1.3)	0.414
Amikacin	673 (0.8)	39 (0.7)	0.393
Streptomycin	5 (0.0)	0 (0.0)	0.564
Kanamycin	523 (0.6)	33 (0.6)	0.758
**Fluoroquinolone**	9,826 (11.8)	1,109 (20.0)	<0.001
Ofloxacin	5,679 (6.8)	623 (11.2)	<0.001
Ciprofloxacin	2,691 (3.2)	281 (5.1)	<0.001
Levofloxacin	2,173 (2.6)	282 (5.1)	<0.001
Moxifloxacin	754 (0.9)	124 (2.2)	<0.001
**Tetracycline**	17,548 (21.1)	1,833 (33.0)	<0.001
Doxycycline	14,150 (17)	1,475 (26.6)	<0.001
Minocycline	5,290 (6.4)	613 (11.0)	<0.001
Tigecycline	10 (0.0)	2 (0.0)	0.136
**Carbapenem**	222 (0.3)	20 (0.4)	0.195
Imipenem	153 (0.2)	13 (0.2)	0.400
Meropenem	86 (0.1)	7 (0.1)	0.611
**Cefoxitin**	289 (0.3)	20 (0.4)	0.871
**Linezolid**	7 (0.0)	0 (0.0)	0.495

Abbreviations: SS, Sjögren’s syndrome; percentages are enclosed in parentheses after each value unless otherwise indicated.

### Association between the use of antibiotics and the risk of Sjögren’s syndrome

Next, we investigated the association between the risk of SS and the use of antibiotics prescribed to treat NTM infection. A conditional logistic regression model that was adjusted for CCI, bronchiectasis and *H. pylori* infection revealed that SS case subjects were more likely to have received new macrolides (aOR 1.95, 95% CI 1.80–2.11), FQNs (aOR 1.52, 95% CI 1.41–1.64) and tetracyclines (aOR 1.69, 95% CI 1.59–1.79) than non-SS control subjects (Table 3, model B). Notably, we found that the association was consistent among each antibiotic in these three groups of antibiotics (Table 3, model A). Among the new macrolides, clarithromycin (aOR 1.84, 95% CI 1.69–2.01) and azithromycin (aOR 2.07, 95% CI 1.71–2.51) had a similar effect on the incident SS. The four FQNs including ofloxacin (aOR 1.43, 95% CI 1.30–1.56), ciprofloxacin (aOR 1.15, 95% CI 1.01–1.32), levofloxacin (aOR 1.34, 95% CI 1.17–1.54), and moxifloxacin (aOR 1.22, 95% CI 1.22–1.85) also had a consistent positive association with incident SS. We found a consistent positive correlation of incident SS with doxycycline (aOR 1.59, 95% CI 1.49–1.70) and minocycline (aOR 1.48, 95% CI 1.35–1.62) but not with tigecycline (aOR 2.03, 95% CI 0.38–10.99), given that very few subjects received tigecycline. In contrast to these three groups of antibiotics, the use of aminoglycosides, especially amikacin, was found to have a negative association with the development of SS (aOR 0.68, 95% CI 0.53–0.87). Taken together, these data showed that the use of new macrolides, FQNs and tetracyclines was consistently associated with the development of SS, whereas aminoglycoside use appeared to be a protective factor.

**Table 3:** Unadjusted and adjusted odds ratios for the association between variables and the risk of Sjogren’s syndrome.

	Univariate analysis	Multivariable A^a^	Multivariable B^a^
	OR (95% CI)	OR (95% CI)	OR (95% CI)
**New macrolide**	2.48 (2.30–2.68)		1.95 (1.80–2.11)
Clarithromycin	2.36 (2.17–2.56)	1.84 (1.69–2.01)	
Azithromycin	3.02 (2.52–3.63)	2.07 (1.71–2.51)	
**Aminoglycoside**	0.90 (0.71–1.15)		0.68 (0.53–0.87)
Amikacin	0.87 (0.63–1.20)	0.58 (0.41–0.81)	
Streptomycin	<0.001 (<0.001–>999)		
Kanamycin	0.95 (0.67–1.35)	0.83 (0.58–1.19)	
**Fluoroquinolone**	1.89 (1.76–2.02)		1.52 (1.41–1.64)
Ofloxacin	1.61 (1.42–1.83)	1.15 (1.01–1.32)	
Ciprofloxacin	2.02 (1.78–2.30)	1.34 (1.17–1.54)	
Levofloxacin	2.52 (2.08–3.06)	1.50 (1.22–1.85)	
Moxifloxacin	1.73 (1.58–1.89)	1.43 (1.30–1.56)	
**Tetracycline**	1.88 (1.78–2.00)		1.69 (1.59–1.79)
Doxycycline	1.81 (1.70–1.92)	1.59 (1.49–1.70)	
Minocycline	1.84 (1.68–2.01)	1.48 (1.35–1.62)	
Tigecycline	3.01 (0.66–13.75)	2.03 (0.38–10.99)	
**Carbapenem**	1.35 (0.86–2.14)		0.87 (0.54–1.39)
Imipenem	1.28 (0.72–2.25)	0.85 (0.47–1.54)	
Meropenem	1.22 (0.57–2.64)	0.72 (0.32–1.64)	
Cefoxitin	1.04 (0.66–1.64)	0.73 (0.46–1.17)	0.74 (0.47–1.18)
Linezolid	<0.001 (<0.001–>999)		
CCI ≥ 1	1.90 (1.78–2.02)	1.61 (1.51–1.72)	1.60 (1.50–1.71)
Bronchiectasis	3.33 (2.85–3.90)	2.30 (1.95–2.71)	2.38 (2.02–2.80)
History of H. pylori infection	3.76 (2.77–5.10)	1.88 (1.36–2.58)	1.80 (1.31–2.47)

^a^Antibiotics in the same group were integrated into model B and seen as independent variables in model A. Abbreviations: OR, odds ratio; CI: confidence intervals; CCI, Charlson comorbidity index; *H. pylori, Helicobacter pylori*.

### Early risk of antibiotic-associated SS

To address the time course of the development of antibiotic-associated SS, we categorized those receiving new macrolides, FQNs, tetracyclines and amikacin into four quarters (Q1–Q4) based on the interval between the use of antibiotics and the diagnosis of SS. We found that the correlation tended to be stronger in Q1 than in Q2, Q3 or Q4 for most antibiotics (Supplementary Tables 2 and 3). Thus, we divided the use of antibiotics into Q1 and Q2–Q4 to address the early correlation between the use of antibiotics and incident SS (Table 4). The multivariate logistic regression showed a consistently higher correlation between the use of antibiotics and the development of SS in Q1 than in Q2-Q4 for clarithromycin (aOR: 1.99 vs. 1.81), azithromycin (aOR: 2.75 vs. 1.83), amikacin (aOR: 0.68 vs. 0.50), ofloxacin (aOR: 1.61 vs. 1.38), ciprofloxacin (aOR: 1.26 vs. 1.11), levofloxacin (aOR: 1.57 vs. 1.26), moxifloxacin (aOR: 2.59 vs. 1.16), doxycycline (aOR: 1.75 vs. 1.54), and minocycline (aOR: 1.70 vs. 1.42). Collectively, these findings identified an early correlation between the use of new macrolides, FQNs, and tetracyclines and the development of SS, whereas amikacin appeared to have a protective role.

**Table 4:** Odds ratios for incident SS of each antibiotic stratified by interval between the initiation of antibiotics and diagnosis of SS^a^.

	Univariate	Multivariable
	OR (95% CI)	OR (95% CI)
**Clarithromycin**		
None used	Reference	Reference
Q1	2.82 (2.44–3.26)	1.99 (1.71–2.33)
Q2–Q4	2.21 (2.01–2.43)	1.81 (1.64–1.99)
**Azithromycin**		
None used	Reference	Reference
Q1	4.46 (3.26–6.10)	2.75 (1.98–3.82)
Q2–Q4	2.56 (2.04–3.20)	1.83 (1.45–2.31)
**Amikacin**		
None used	Reference	Reference
Q1	1.34 (0.79–2.27)	0.68 (0.39–1.19)
Q2–Q4	0.71 (0.47–1.07)	0.50 (0.33–0.76)
**Ofloxacin**		
None used	Reference	Reference
Q1	1.96 (1.67–2.30)	1.61 (1.37–1.90)
Q2–Q4	1.65 (1.49–1.83)	1.38 (1.24–1.53)
**Ciprofloxacin**		
None used	Reference	Reference
Q1	1.81 (1.43–2.29)	1.26 (0.99–1.62)
Q2–Q4	1.54 (1.33–1.79)	1.11 (0.95–1.30)
**Levofloxacin**		
None used	Reference	Reference
Q1	2.51 (2.00–3.16)	1.57 (1.23–1.99)
Q2–Q4	1.86 (1.60–2.17)	1.26 (1.07–1.48)
**Moxifloxacin**		
None used	Reference	Reference
Q1	4.46 (3.24–6.15)	2.59 (1.84–3.64)
Q2–Q4	1.97 (1.54–2.51)	1.16 (0.89–1.49)
**Doxycycline**		
None used	Reference	Reference
Q1	1.96 (1.76–2.19)	1.75 (1.57–1.96)
Q2–Q4	1.75 (1.63–1.88)	1.54 (1.43–1.66)
**Minocycline**		
None used	Reference	Reference
Q1	2.08 (1.77–2.45)	1.70 (1.44–2.01)
Q2–Q4	1.76 (1.59–1.95)	1.42 (1.28–1.58)
CCI ≥ 1	1.90 (1.78–2.02)	1.59 (1.49–1.70)
Bronchiectasis	3.33 (2.85–3.90)	2.32 (1.96–2.74)
History of H. pylori infection	3.76 (2.77–5.10)	1.79 (1.29–2.49)

^a^Interval between the use of antibiotics and the diagnosis of SS was stratified into four quarters, namely, Q1, Q2, Q3 and Q4, and Q2–Q4 were integrated into one variable. Abbreviations: OR, odds ratio; CI: confidence intervals; CCI, Charlson comorbidity index; *H. pylori, Helicobacter pylori*.

## Discussion

To our knowledge, the present study is the first to investigate the association between the use of antibiotics for NTM infection and the risk of SS using a population-based dataset. Our results demonstrated that the use of new macrolides, FQNs and tetracyclines, which are frequently used to treat a wide range of infectious diseases, was positively associated with the development of SS, whereas the use of amikacin appeared to play a protective role. These findings indicate the need for vigilance in prescribing these antibiotics to treat not only NTM infection but also other infectious diseases.

Early recognition of the adverse effects of antibiotics is difficult, particularly in treating NTM infections that require combined conventional anti-mycobacterial agents and other antibiotics^9^. Among these antibiotics, the constitutional symptoms of NTM infection itself and adverse effects, particularly hepatotoxicity and gastrointestinal intolerance of conventional anti-mycobacterial agents, may lead to the delayed recognition of antibiotic-associated SS, which has an insidious onset and nonspecific symptoms. Given that we aimed to investigate the distinct role of antibiotic-associated SS among antimicrobials other than conventional anti-mycobacterial agents, such as isoniazid, ethambutol, rifampicin, and pyrazinamide, patients who had been diagnosed with a mycobacterial infection were thus excluded. We thought that this approach would enable us to specifically determine the association between the use of FQNs, tetracyclines and new macrolides and incident SS, as shown in this study. Furthermore, we also tested the potential interaction effects among antibiotics and conducted a total of 9 subgroup analyses to validate the independent association between the use of FQNs, tetracyclines, and new macrolides and incident SS (Supplemental Tables 5–7). Additionally, the Bonferroni correction, a stringent method to reduce potential type I error in potential multiple comparisons, also showed similar associations in the 8 subgroup analyses although mildly impaired strength in one subgroup (Supplemental Tables 8). Similar results were found in the multivariate analysis of individual antibiotics after Bonferroni correction (Supplemental Tables 9). We believe that these findings have crucial clinical impact given that these antibiotics have been used in a wide range of infectious diseases, including urinary tract infection, pneumonia, acne, and *H. pylori* infection.

ADRs consists of intrinsic type (type A) and idiosyncratic type (type B) reactions^1,2,10^. DIA is one of the idiosyncratic ADRs; however, the exact incidence of DIA remains unclear due to the difficulties in clarifying the causality of DIA, except for the diagnosis of drug-induced lupus, which is largely supported by the presence of anti-histone autoantibodies^4,5^. It is estimated that 5 per 10,000 of those taking minocycline develop lupus through binding to tissue macromolecules, which leads to immunogenic cross-reactivity^11^, and HLA-DQB1, HLA-DR2 or HLA-DR4 have been implicated in minocycline-induced lupus^12^. Additionally, minocycline-induced lupus mostly resolves after drug discontinuation, while a substantial proportion of young children may have prolonged disease following minocycline exposure^13^. There are numerous reports of minocycline-induced lupus in the literature, but to date, few studies on minocycline-associated SS have been conducted. In this study, we found that the use of minocycline was independently associated with the development of SS. Notably, consistent with previous studies on minocycline-induced lupus^14,15^, acne was also the major indication for minocycline in the present study. These findings provide real-world practical evidence and highlight the need for vigilance among practitioners given that patients with acne are generally treated at clinics.

FQNs and new macrolides are the two fundamental classes of antibiotics used in the treatment of NTM infection. FQNs have a wide range of clinical indications; however, adverse effects are not unusual for FQNs. The most commonly reported FQN-associated adverse effects are gastrointestinal and central nervous system toxicities, while other uncommon adverse effects include skin rashes, tendinitis, QT prolongation, hypoglycaemia, hyperglycaemia, and haematologic toxicity^16,17^. Interestingly, data regarding FQN-associated autoimmunity and reports of autoimmune haemolytic anaemia are extremely scarce^18,19^, and we postulated that FQN-associated autoimmunity might be potentially underestimated due to the insidious onset and the nonspecific clinical manifestations of autoimmune diseases, including SS. In this study, we found a consistent positive association between incident SS and the use of the four FQNs including ofloxacin, ciprofloxacin, levofloxacin and moxifloxacin. This finding is of considerable clinical relevance given that FQNs are administered for a wide range of infectious diseases.

Like FQNs, new macrolides constitute another crucial antibiotic class administered for NTM infection. Clarithromycin, the main new macrolide used to treat NTM infection, has been implicated in drug-drug interactions by inhibiting cytochrome P450 3A4^20^. Moreover, new macrolides have been reported to aggravate experimental autoimmune encephalomyelitis by inhibiting the production of nitric oxide^21^, indicating that new macrolides might exert autoimmunity directly through the nitric oxide pathway. Notably, we found that the main indication for macrolides including clarithromycin and azithromycin in the present study was to treat *H. pylori* infection, which is consistent with one recently published report on real-world practice to eradicate *H. pylori* infection with clarithromycin or azithromycin in Asia-Pacific countries^22^. Given that *H. pylori* infection remains an ongoing challenge in Asian countries^23^, our results shed light on the need for vigilance with potential new macrolide-associated SS in treating not only patients with NTM infection but also those with *H. pylori* infection.

Interestingly, in contrast to the positive association between the use of new macrolides, FQNs and tetracyclines and incident SS, the use of amikacin was negatively correlated with the development of SS. The three aminoglycosides, namely, amikacin, streptomycin and kanamycin, which are used to treat mycobacterial infection have a similar antimicrobial spectrum^9,24^, whereas such a protective role of amikacin in the development of SS was not found for the other two aminoglycosides. Therefore, we postulate that the protective effect of amikacin against incident SS might be attributed to a distinct non-antibiotic effect of amikacin. For example, amikacin has a distinct post-antibiotic effect against *Pseudomonas aeruginosa* and *Staphylococcus aureus*, although the underlying mechanisms have not yet been elucidated^25,26^.

Our previous study suggested that NTM infection may be implicated in the development of SS, possibly through dysregulated inflammation^8,27^. In the present study, the association between NTM infection and the risk of SS remained robust after adjusting for the usage of antibiotics (aOR 12.55, 95% CI 2.23–70.75), and we noted that *H. pylori* was weakly associated with the risk of SS (aOR 1.71, 95% CI 1.27–2.32) (Supplementary Table 4). Indeed, the positive association between *H. pylori* infection and SS has been shown in a number of autoimmune diseases^28,29^, and these findings, including our studies, support the notion that autoimmune diseases may arise from the interaction of genetic susceptibility and environmental exposures including infectious triggers^30^.

There are limitations in this study that merit discussion. First, we excluded those with a history of mycobacterial infection to clarify the independent effect of antibiotics on incident SS; however, the correlation remained consistent in the analysis that included individuals with a history of NTM infection (Supplementary Table 4). Second, similar to the current evidence regarding drug-induced autoimmunity, we could not assess the causality of antibiotic-associated SS in this study. Thus, we discussed antibiotic-associated SS rather than antibiotic-induced SS, and the findings of this study merit further mechanistic studies. Third, the accuracy of diagnoses according to claims data is of concern. However, regular quality control evaluations of claims data from all medical institutions by the BNHI has improved coding accuracy^31^, and bias resulting from misclassification was thus minimized. Fourth, viral infections, including cytomegalovirus and Epstein–Barr virus infection, have been implicated in incident SS^32,33^; however, the accurate diagnosis of viral infections could not be assessed in claim data.

In conclusion, we found that several antibiotics used to treat NTM infection were associated with incident SS using a nationwide, population-based dataset. The use of new macrolides, FQNs and tetracyclines was positively correlated with incident SS, whereas amikacin may be negatively associated with incident SS. This study supports the need for vigilance of SS in patients treated with antibiotics for NTM and other infections, including acne, urinary tract infection, pneumonia, and *H. pylori* infection. More mechanistic studies are warranted in the future to explore the underlying mechanisms.

## Methods

### Ethical statements

This study was approved by the Institutional Review Board of Taichung Veterans General Hospital, Tai-wan (IRB number: CE16251A). Informed consent was not required given that the NHIRD data files contain only de-identified secondary data.

### Study design

This study was a retrospective, matched case-control study.

### Data source

In Taiwan, a single-payer National Health Insurance (NHI) program was launched in 1995 and has wide coverage, with 99.6% of Taiwan’s population enrolled in 2015^34^. The National Health Insurance Research Database (NHIRD) is the database of the NHI program containing the registration profiles and original claims data for reimbursement. In the present study, we used the ambulatory, inpatient, and enrolment data from the 2003–2012 NHIRD to identify subjects with newly diagnosed SS. Moreover, in Taiwan, patients with certain major illnesses, including cancer and certain autoimmune diseases, including SS, are classified as having “catastrophic illness” and are therefore exempted from co-payment. The diagnosis of SS in Taiwan is in accordance with the classification criteria for SS proposed by the American–European Consensus Group in 2002^35^. Briefly, subjects with SS are issued a certificate of catastrophic illness for SS if two qualified rheumatologists validate their SS diagnosis after a full review of medical records, laboratory data, pathological data, and images. Furthermore, the NHIRD also contains a catastrophic illness enrolment profile for subjects with catastrophic illness certificates, so-called the Registry for Catastrophic Illness Patient Database (RCIPD). In this study, we enrolled SS patients whose details were found in the RCIPD. The NHIRD also constructed a representative database of 1,000,000 individuals via random selection from all enrolees who received services in 2000 (Longitudinal Health Insurance Database, LHID2000). In this study, the data of the non-SS control subjects were extracted by matching SS cases for age, gender, and the year of first SS diagnosis from the LHID2000 database.

### Diagnosis and anti-mycobacterial antibiotics for mycobacterial infection

Mycobacterial infection was identified using the International Classification of Diseases, 9th Revision, Clinical Modification (ICD-9-CM) codes for tuberculosis (TB): 010–018 and NTM: 031.0, 031.1, 031.2, 031.8, and 031.9 with the concurrent administration of at least two anti-mycobacterial drugs within 12 months of the diagnosis. Anti-mycobacterial antibiotics prior to the diagnosis of SS consisted of conventional anti-mycobacterial medications (isoniazid, ethambutol, rifampicin, and pyrazinamide), new macrolides (clarithromycin and azithromycin), fluoroquinolones (FQNs) (ofloxacin, ciprofloxacin, levofloxacin, and moxifloxacin), aminoglycosides (streptomycin, amikacin, and kanamycin), tetracyclines (minocycline, doxycycline, and tigecycline), carbapenems (imipenem and meropenem), cefoxitin, and linezolid^9^.

### Study subjects

In the present study, SS patients were identified as having either at least three ambulatory visits or one hospital admission with a diagnosis of SS (ICD-9-CM code 710.2) and a catastrophic illness certificate for SS. Given that we aimed to identify patients with newly diagnosed SS, those who had an SS diagnosis before 2007 were thus excluded. To avoid the inclusion of subjects with secondary SS resulting from rheumatoid arthritis (RA) or systemic lupus erythaematosus (SLE), we also excluded individuals who ever had a diagnosis of RA (ICD-9-CM code 714.0) or SLE (ICD-9-CM code 710.0) before the index date; thus, all the enrolled SS patients were newly diagnosed SS cases. The index date of SS patients was defined as the first date of visits with an SS diagnosis, and the index year was the year of the index date. Matched non-SS subjects were randomly selected from one million representative populations from the LHID2000, matching SS cases (1:15) for age, gender, and the index year after the exclusion of individuals who ever had ICD-9 codes for NTM, TB, or diseases of connective tissue (ICD-9-CM codes 710.x) during 2003–2012. The index date used for non-SS controls was the day of the first ambulatory visit for any reason in the index year.

### Potential confounders

Potential confounders included in the adjustment in the logistical regression model included age, gender, Charlson comorbidity index (CCI) (0, ≥ 1), bronchiectasis, and *Helicobacter pylori (H. pylori)* infection. The presence of comorbidity was defined as having at least three ambulatory visits or one inpatient visit with a corresponding ICD-9CM code within 1 year before the index date. The CCI, as adapted by Deyo *et al*.,^36^ was used to evaluate the level of general comorbid medical conditions. Given that *H. pylori* infection was one of main indications for clarithromycin and *H. pylori* infection has been implicated in autoimmune diseases^37,38^, we defined *H. pylori* infection within 1 year before the index date as a potential confounder in this study. Additionally, bronchiectasis has been reported to be associated with NTM infection and SS, and we thus considered bronchiectasis, defined by having either one hospital admission or at least three ambulatory visits with a diagnosis of bronchiectasis (ICD-9-CM code 494.x), as a potential confounder^39,40^.

### Statistical analysis

Data were presented as the mean ± standard deviation (SD) for continuous variables and as numbers (percentages) for categorical variables. The differences were analysed using Student’s t-test for continuous variables and Pearson’s *χ*^2^ test for categorical variables. A conditional logistical regression model was conducted to estimate the odds ratio (OR) of newly diagnosed SS after adjustment for age, gender, history of bronchiectasis, CCI, and history of *H. pylori* infection. The significance of interaction effects between covariates on the risk of SS was investigated by calculating the P-value of the coefficient associated with the product of the indicators of the covariates using the Wald test. Additionally, Bonferroni correction was used due to the potential for multiple comparisons. All the data were analysed using SPSS statistical software version 9.3 (SAS Institute, Inc., Cary, NC, USA). A P value < 0.05 was considered statistically significant.

## Electronic supplementary material

Supplemental data

## Data Availability

All data generated or analysed during this study are included in this article and its Supplementary Information files.
