# Supplementary material for: Associations between Antibiotics for Non-tuberculous Mycobacterial Infection and Incident Sjögren’s Syndrome: A Nationwide, Population-based Case-control Study
Source: Sci Rep. 2018 Oct 30;8:16007. doi: 10.1038/s41598-018-34495-4 (PMC6207743; doi:10.1038/s41598-018-34495-4)
Supplement: Supplementary file 1 — Supplemental data [file 41598_2018_34495_MOESM1_ESM.pdf]

Associations between Antibiotics for Non-tuberculous Mycobacterial Infection and Incident Sjögren's Syndrome: A Nationwide, Population-based Case-control Study

Wen-Cheng Chao, Ching-Heng Lin, Yi-Ming Chen, Chiann-Yi Hsu, Jun-Peng Chen, Hsin-Hua Chen

Supplementary table 1. Major indications for usage of antibiotics

	N	1st indication	2nd indication	3rd indication
**New macrolide**				
Clarithromycin	6,067	Peptic ulcer/*H. pylori* (3,543)	Bronchitis[a] (1,058)	Pneumonia (382)
Azithromycin	855	Pneumonia (336)	Bronchitis[a] (255)	Respiratory infection[b] (64)
**Aminoglycoside**				
Amikacin	712	UTI (174)	Septicemia (78)	Pneumonia (63)
Streptomycin	5	Pyelonephritis/cystitis (5)	NA	NA
Kanamycin	556	UTI (80)	Respiratory infection[b] (64)	Bronchitis[a] (47)
**Fluoroquinolone**				
Ofloxacin	6,302	Respiratory infection[b] (791)	UTI (735)	Sinusitis (702)
Ciprofloxacin	2,972	UTI (718)	Pyelonephritis/cystitis (254)	Septicemia (99)
Levofloxacin	2,455	UTI (664)	Pneumonia (390)	Pyelonephritis/cystitis (137)
Moxifloxacin	878	Pneumonia (338)	Bronchitis[a] (150)	UTI (60)
**Tetracycline**				
Doxycycline	15,625	Acne (3,235)	Sinusitis (1,551)	Vaginitis/Vulvovaginitis (1,476)
Minocycline	5,903	Sinusitis (1,852)	Bronchitis[a] (1,049)	Acne (922)
Tigecycline	12	Pneumonia (5)	UTI (4)	Septicemia (2)

**Carbapenem**				
Imipenem	166	UTI (36)	Septicemia (35)	Pneumonia (25)
Meropenem	93	Pneumonia (29)	UTI (29)	Septicemia (18)
**Cefoxitin**	309	UTI (82)	Septicemia (33)	Pneumonia (25)
**Linezolid**	7	Pneumonia (3)	Soft tissue infection (2)	Septicemia (1)

[a] Bronchitis included bronchitis (ICD: 466.0) and bronchopneumonia (ICD: 485). [b] Upper respiratory infections of unspecified site (ICD: 465.9). Abbreviation: *H. pylori*, *Helicobacter pylori*; UTI, urinary tract infection; NA, not applicable.

Supplementary table 2. Odds ratios for the associations between each antibiotic and incident SS stratified by interval (quarters) between initiation of antibiotics and diagnosis of SS[a]

	Univariate	Multivariable A[a]
	OR (95% CI)	OR (95% CI)
**Clarithromycin**		
None used	Reference	Reference
Q1	2.82 (2.44–3.26)	2.00 (1.71–2.33)
Q2	2.00 (1.69–2.36)	1.65 (1.39–1.96)
Q3	1.81 (1.53–2.15)	1.49 (1.25–1.77)
Q4	2.86 (2.47–3.31)	2.31 (1.99–2.69)
**Azithromycin**		
None used	Reference	Reference
Q1	4.46 (3.26–6.10)	2.77 (1.99–3.85)
Q2	2.58 (1.75–3.80)	1.77 (1.18–2.63)
Q3	2.99 (2.07–4.33)	2.44 (1.67–3.57)
Q4	2.13 (1.41–3.22)	1.41 (0.92–2.15)
**Amikacin**		
None used	Reference	Reference
Q1	1.34 (0.79–2.27)	0.68 (0.39–1.20)
Q2	0.45 (0.18–1.09)	0.28 (0.11–0.68)
Q3	0.71 (0.35–1.43)	0.54 (0.26–1.12)
Q4	0.99 (0.54–1.82)	0.74 (0.40–1.38)
**Ofloxacin**		
None used	Reference	Reference
Q1	1.96 (1.67–2.31)	1.61 (1.37–1.90)
Q2	1.28 (1.06–1.55)	1.06 (0.88–1.29)
Q3	1.59 (1.34–1.89)	1.35 (1.13–1.61)
Q4	2.11 (1.81–2.47)	1.73 (1.47–2.03)
**Ciprofloxacin**		
None used	Reference	Reference
Q1	1.81 (1.43–2.29)	1.26 (0.98–1.61)
Q2	1.74 (1.37–2.20)	1.29 (1.01–1.66)
Q3	1.47 (1.14–1.90)	1.09 (0.83–1.42)
Q4	1.41 (1.08–1.83)	0.97 (0.74–1.28)
**Levofloxacin**		
None used	Reference	Reference
Q1	2.51 (2.00–3.16)	1.57 (1.23–2.00)
Q2	1.93 (1.49–2.48)	1.36 (1.04–1.77)
Q3	1.96 (1.51–2.53)	1.34 (1.02–1.76)
Q4	1.71 (1.31–2.23)	1.07 (0.81–1.42)
**Moxifloxacin**		
None used	Reference	Reference
Q1	4.46 (3.24–6.15)	2.59 (1.84–3.64)
Q2	1.83 (1.20–2.80)	1.01 (0.65–1.58)
Q3	2.07 (1.37–3.13)	1.30 (0.84–1.99)
Q4	2.00 (1.32–3.04)	1.14 (0.74–1.78)
**Doxycycline**		

None used	Reference	Reference
Q1	1.96 (1.76–2.19)	1.76 (1.57–1.96)
Q2	1.64 (1.46–1.85)	1.50 (1.33–1.69)
Q3	1.77 (1.58–1.98)	1.54 (1.38–1.73)
Q4	1.85 (1.65–2.06)	1.57 (1.40–1.76)
**Minocycline**		
None used	Reference	Reference
Q1	2.08 (1.77–2.45)	1.71 (1.45–2.02)
Q2	1.61 (1.35–1.92)	1.35 (1.13–1.62)
Q3	1.99 (1.68–2.35)	1.61 (1.35–1.91)
Q4	1.70 (1.43–2.03)	1.31 (1.10–1.57)
**CCI≥1**	1.90 (1.78–2.02)	1.60 (1.49–1.71)
**Bronchiectasis**	3.33 (2.85–3.90)	2.33 (1.98–2.75)
**History of *H. pylori***	3.76 (2.77–5.10)	1.78 (1.28–2.47)

[a] Interval between usage of antibiotics and diagnosis of SS was stratified into four quarters, namely Q1, Q2, Q3, and Q4. Abbreviations: OR, odds ratio; CI: confidence intervals; CCI, Charlson comorbidity index; *H. pylori, Helicobacter pylori.*

Supplementary table 3. Interval (months) between usage of antibiotics and diagnosis of SS

	**Interval (months)**
	**(*n* =5,553)**
**New macrolide**	
Clarithromycin	38.4 (51.2)
Azithromycin	25.1 (44.8)
**Aminoglycoside**	
Amikacin	35.9 (56.9)
Streptomycin	NA
Kanamycin	55.9 (39.6)
**Fluoroquinolone**	
Ofloxacin	49.7 (55.1)
Ciprofloxacin	27.9 (39.0)
Levofloxacin	26.4 (38.4)
Moxifloxacin	17.3 (37.0)
**Tetracycline**	
Doxycycline	52.9 (47.3)
Minocycline	51.9 (47.7)
Tigecycline	8.6 (14.5)
**Carbapenem**	
Imipenem	4.0 (10.4)
Meropenem	
**Cefoxitin**	15.0 (37.4)
**Linezolid**	NA

Data were presented with median (interquartile range, IQR)

Supplementary table 4. Odds ratios for the association between variables and the risk of Sjogren's syndrome including those having history of mycobacterial infection

	Univariate analysis OR (95% CI)	Multivariable A[a] OR (95% CI)	Multivariable B[a] OR (95% CI)
**Isoniazid**	1.71 (1.30–2.25)	0.71 (0.33–1.56)	0.74 (0.34–1.63)
**Ethambutol**	1.77 (1.34–2.33)	0.80 (0.31–2.04)	0.64 (0.24–1.69)
**Rifampin**	1.35 (1.10–1.66)	0.77 (0.57–1.05)	0.75 (0.55–1.03)
**Pyrazinamide**	1.87 (1.39–2.51)	1.79 (0.75–4.23)	2.31 (0.91–5.85)
**New macrolide**	2.61 (2.42–2.81)		2.06 (1.90–2.23)
Clarithromycin	2.50 (2.31–2.71)	1.96 (1.80–2.13)	
Azithromycin	3.04 (2.56–3.61)	2.08 (1.74–2.49)	
**Aminoglycoside**	0.88 (0.70–1.10)		0.64 (0.51–0.81)
Amikacin	0.78 (0.57–1.07)	0.53 (0.38–0.74)	
Streptomycin	3.41 (1.29–9.00)	1.31 (0.46–3.76)	
Kanamycin	0.92 (0.65–1.29)	0.78 (0.55–1.10)	
**Fluoroquinolone**	1.86 (1.74–1.99)		1.49 (1.39–1.60)
Ofloxacin	1.71 (1.57–1.86)	1.43 (1.31–1.57)	
Ciprofloxacin	1.53 (1.35–1.73)	1.08 (0.95–1.24)	
Levofloxacin	2.01 (1.78–2.27)	1.34 (1.17–1.53)	
Moxifloxacin	2.48 (2.09–2.96)	1.47 (1.22–1.78)	
**Tetracycline**	1.95 (1.84–2.06)		1.75 (1.65–1.86)
Doxycycline	1.87 (1.76–1.99)	1.65 (1.55–1.76)	
Minocycline	1.86 (1.70–2.03)	1.49 (1.36–1.63)	
Tigecycline	2.39 (0.70–8.13)	2.00 (0.52–7.64)	
**Carbapenem**	1.15 (0.73–1.81)		0.76 (0.47–1.21)
Imipenem	1.12 (0.63–1.96)	0.72 (0.40–1.31)	
Meropenem	0.97 (0.45–2.09)	0.63 (0.28–1.44)	
**Cefoxitin**	0.98 (0.64–1.52)	0.68 (0.43–1.07)	0.69 (0.44–1.07)
**Linezolid**	<0.001 (-)		
**Mycobacterial infection**[b]	1.98 (1.51–2.59)	1.44 (0.66–3.13)	
NTM	20.00 (4.48–89.36)		12.55 (2.23–70.75)
TB	1.86 (1.41–2.45)		1.40 (0.63–3.10)
**CCI≥1**	1.89 (1.77–2.01)	1.58 (1.48–1.69)	1.57 (1.47–1.68)
**Bronchiectasis**	3.19 (2.76–3.69)	2.08 (1.78–2.43)	2.14 (1.84–2.50)
**History of *H. pylori* infection**	3.69 (2.76–4.94)	1.79 (1.32–2.42)	1.71 (1.27–2.32)

[a]Antibiotics in the same group were integrated into model B and seen as independent variables in model A. [b]Confirmed infection. Abbreviations: OR, odds ratio; CI: confidence intervals; CCI, Charlson comorbidity index; *H. pylori*, *Helicobacter pylori.*

Supplemental table 5. Analysis of the interaction effects among different antibiotic groups on the risk of incident Sjogren's syndrome

	All patients		Subgroups analyses					
	3-factor model	2-factor model	New macrolide		Fluoroquinolone		Tetracycline	
			No	Yes	No	Yes	No	Yes
**New macrolide**	<0.001	<0.001			<0.001	<0.001	<0.001	<0.001
**Fluoroquinolone**	<0.001	<0.001	<0.001	0.001			<0.001	<0.001
**Tetracycline**	<0.001	<0.001	<0.001	<0.001	<0.001	<0.001		
**New macrolide*Fluoroquinolone**	0.053	0.021					0.006	0.811
**Fluoroquinolone*Tetracycline**	0.464	0.042	0.014	0.827				
**New macrolide*Tetracycline**	0.156	0.023			0.008	0.787		
**New macrolide*Fluoroquinolone*Tetracycline**	0.133							

Adjusted for usage of aminoglycosides, carbapenem, cefoxitin, Charlson comorbidity index, bronchiectasis, and history of *Helicobacter pylori*.

Supplemental table 6. Adjusted odds ratios for the association between variables and the risk of Sjogren's syndrome in the subgroups of using new macrolide, fluoroquinolone or tetracycline

	New macrolide	Fluoroquinolone	Tetracycline
	Yes	Yes	Yes
	OR (95% CI)	OR (95% CI)	OR (95% CI)
**New macrolide**		1.81 (1.55–2.12)	1.71 (1.49–1.96)
**Fluoroquinolone**	1.34 (1.14–1.59)		1.38 (1.23–1.56)
**Tetracycline**	1.36 (1.16–1.59)	1.41 (1.23–1.60)	
**Aminoglycoside**	0.77 (0.46–1.30)	0.50 (0.33–0.75)	0.61 (0.41–0.91)
**Carbapenem**	1.56 (0.72–3.41)	0.82 (0.46–1.48)	1.04 (0.48–2.24)
**Cefoxitin**	0.74 (0.31–1.77)	0.58 (0.28–1.19)	1.50 (0.78–2.89)
**CCI ≥1**	1.39 (1.18–1.63)	1.15 (0.99–1.33)	1.59 (1.41–1.79)
**Bronchiectasis**	2.58 (1.91–3.49)	2.54 (1.95–3.32)	1.90 (1.43–2.52)
**History of *H. pylori***	1.73 (1.21–2.49)	2.13 (1.17–3.88)	1.82 (0.99–3.33)

Abbreviations: OR, odds ratio; CI: confidence intervals; CCI, Charlson comorbidity index; *H. pylori*, *Helicobacter pylori*.

Supplemental table 7. Adjusted odds ratios for the association between variables and the risk of Sjogren's syndrome in the subgroups based on antibiotic-pair with a potential interaction effect

	New macrolide No Fluoroquinolone No OR (95% CI)	New macrolide No Fluoroquinolone Yes OR (95% CI)	New macrolide No Tetracycline No OR (95% CI)	New macrolide No Tetracycline Yes OR (95% CI)	Fluoroquinolone No New macrolide Yes OR (95% CI)	Fluoroquinolone No Tetracycline No OR (95% CI)	Fluoroquinolone No Tetracycline Yes OR (95% CI)	Tetracycline No New macrolide Yes OR (95% CI)	Tetracycline No Fluoroquinolone Yes OR (95% CI)
**New macrolide**						2.22 (1.98–2.49)	1.71 (1.45–2.02)		1.80 (1.46–2.23)
**Fluoroquinolone**			1.71 (1.55–1.90)	1.37 (1.20–1.57)				1.28 (1.03–1.59)	
**Tetracycline**	1.83 (1.70–1.97)	1.42 (1.22–1.65)			1.38 (1.15–1.67)				
**Aminoglycoside**	0.93 (0.67–1.29)	0.40 (0.24–0.68)	0.75 (0.54–1.05)	0.50 (0.30–0.85)	0.82 (0.38–1.75)	1.03 (0.71–1.47)	0.74 (0.43–1.29)	0.51 (0.21–1.21)	0.40 (0.21–0.74)
**Carbapenem**	0.62 (0.15–2.57)	0.82 (0.41–1.65)	0.51 (0.22–1.16)	0.87 (0.34–2.25)	6.17 (1.78–21.34)	2.12 (0.81–5.53)	1.21 (0.27–5.42)	1.65 (0.63–4.30)	0.67 (0.30–1.48)
**Cefoxitin**	1.06 (0.56–2.03)	0.45 (0.16–1.23)	0.37 (0.15–0.91)	1.63 (0.80–3.36)	0.64 (0.14–2.88)	0.68 (0.30–1.56)	1.51 (0.61–3.72)	0.59 (0.20–1.74)	0.30 (0.09–0.97)
**CCI ≥1**	1.80 (1.66–1.95)	1.18 (1.002–1.39)	1.66 (1.52–1.81)	1.62 (1.41–1.85)	1.52 (1.26–1.83)	1.79 (1.64–1.95)	1.69 (1.47–1.94)	1.38 (1.14–1.69)	1.02 (0.85–1.23)
**Bronchiectasis**	2.46 (1.94–3.12)	2.37 (1.69–3.33)	2.50 (1.97–3.18)	2.24 (1.60–3.14)	2.34 (1.54–3.55)	2.63 (2.05–3.38)	2.01 (1.40–2.90)	3.66 (2.52–5.31)	2.97 (2.14–4.13)
**History of *H. pylori***	2.91 (1.42–5.96)	1.29 (0.15–10.92)	3.80 (1.83–7.87)	0.72 (0.09–5.46)	1.61 (1.03–2.51)	1.61 (1.03–2.51)	1.80 (0.88–3.68)	1.68 (1.08–2.61)	2.28 (1.12–4.65)

Abbreviations: OR, odds ratio; CI: confidence intervals; CCI, Charlson comorbidity index; *H. pylori*, *Helicobacter pylori*.

Supplemental table 8. Bonferroni corrected adjusted odds ratios for the association between variables and the risk of Sjogren's syndrome [a]

	New macrolide	New macrolide	New macrolide	New macrolide	Fluoroquinolone	Fluoroquinolone	Fluoroquinolone	Tetracycline	Tetracycline
	**No**	**No**	**No**	**No**	**No**	**No**	**No**	**No**	**No**
	**Fluoroquinolone**	**Fluoroquinolone**	**Tetracycline**	**Tetracycline**	**New macrolide**	**Tetracycline**	**Tetracycline**	**New macrolide**	**Fluoroquinolone**
	**No**	**Yes**	**No**	**Yes**	**Yes**	**No**	**Yes**	**Yes**	**Yes**
	**OR (98.75% CI)**	**OR (98.75% CI)**	**OR (98.75% CI)**	**OR (98.75% CI)**	**OR (98.75% CI)**	**OR (98.75% CI)**	**OR (98.75% CI)**	**OR (98.75% CI)**	**OR (98.75% CI)**
**New macrolide**						2.22 (1.92–2.57)	1.71 (1.38–2.11)		1.80 (1.37–2.36)
**Fluoroquinolone**			1.71 (1.51–1.95)	1.37 (1.16–1.63)				1.28 (0.97–1.69)	
**Tetracycline**	1.83 (1.67–2.01)	1.42 (1.17–1.72)			1.38 (1.09–1.76)				
**Aminoglycoside**	0.93 (0.61–1.42)	0.40 (0.21–0.79)	0.75 (0.49–1.15)	0.50 (0.26–0.98)	0.82 (0.31–2.15)	1.03 (0.65–1.63)	0.74 (0.37–1.50)	0.51 (0.17–1.53)	0.40 (0.18–0.87)
**Carbapenem**	0.62 (0.10–3.81)	0.82 (0.34–2.00)	0.51 (0.18–1.45)	0.87 (0.26–2.92)	6.17 (1.27–30.01)	2.12 (0.62–7.20)	1.21 (0.18–8.18)	1.65 (0.48–5.60)	0.67 (0.24–1.84)
**Cefoxitin**	1.06 (0.47–2.43)	0.45 (0.12–1.62)	0.37 (0.12–1.17)	1.63 (0.65–4.09)	0.64 (0.09–4.35)	0.68 (0.24–1.97)	1.51 (0.48–4.77)	0.59 (0.15–2.33)	0.30 (0.07–1.34)
**CCI ≥1**	1.80 (1.62–1.99)	1.18 (0.96–1.46)	1.66 (1.49–1.86)	1.62 (1.36–1.92)	1.52 (1.20–1.92)	1.79 (1.60–2.00)	1.69 (1.41–2.02)	1.38 (1.08–1.78)	1.02 (0.81–1.29)
**Bronchiectasis**	2.46 (1.81–3.33)	2.37 (1.54–3.65)	2.50 (1.85–3.40)	2.24 (1.46–3.44)	2.34 (1.37–3.98)	2.63 (1.91–3.62)	2.01 (1.26–3.20)	3.66 (2.27–5.89)	2.97 (1.95–4.52)
**History of *H. pylori***	2.91 (1.16–7.27)	1.29 (0.09–19.61)	3.80 (1.50–9.61)	0.72 (0.05–9.53)	1.61 (0.91–2.84)	1.61 (0.91–2.84)	1.80 (0.72–4.48)	1.68 (0.96–2.95)	2.28 (0.92–5.65)

[a]The 98.75% CI reflected that the set type I error was 0.05 divided by 4 given that 4 antibiotic groups might be used in each subgroup.

Abbreviations: OR, odds ratio; CI: confidence intervals; CCI, Charlson comorbidity index; *H. pylori*, *Helicobacter pylori*.

Supplemental table 9. Odds ratios with and without Bonferroni correction for the association between variables and incident Sjogren's syndrome

	Univariate analysis	Multivariate	Multivariate [a]
	OR (95% CI)	OR (95% CI)	OR (99.64% CI)
**New macrolide**	2.48 (2.30–2.68)		
Clarithromycin	2.36 (2.17–2.56)	1.84 (1.69–2.01)	1.84 (1.62–2.09)
Azithromycin	3.02 (2.52–3.63)	2.07 (1.71–2.51)	2.07 (1.56–2.75)
**Aminoglycoside**	0.90 (0.71–1.15)		
Amikacin	0.87 (0.63–1.20)	0.58 (0.41–0.81)	0.58 (0.35–0.95)
Streptomycin	<0.001		
Kanamycin	0.95 (0.67–1.35)	0.83 (0.58–1.19)	0.83 (0.49–1.42)
**Fluoroquinolone**	1.89 (1.76–2.02)		
Ofloxacin	1.61 (1.42–1.83)	1.15 (1.01–1.32)	1.15 (0.95–1.41)
Ciprofloxacin	2.02 (1.78–2.30)	1.34 (1.17–1.54)	1.34 (1.09–1.65)
Levofloxacin	2.52 (2.08–3.06)	1.50 (1.22–1.85)	1.50 (1.10–2.04)
Moxifloxacin	1.73 (1.58–1.89)	1.43 (1.30–1.56)	1.43 (1.25–1.63)
**Tetracycline**	1.88 (1.78–2.00)		
Doxycycline	1.81 (1.70–1.92)	1.59 (1.49–1.70)	1.59 (1.45–1.76)
Minocycline	1.84 (1.68–2.01)	1.48 (1.35–1.62)	1.48 (1.29–1.70)
Tigecycline	3.01 (0.66–13.75)	2.03 (0.38–10.99)	2.03 (0.17–24.91)
**Carbapenem**	1.35 (0.86–2.14)		
Imipenem	1.28 (0.72–2.25)	0.85 (0.47–1.54)	0.85 (0.35–2.05)
Meropenem	1.22 (0.57–2.64)	0.72 (0.32–1.64)	0.72 (0.21–2.45)
**Cefoxitin**	1.04 (0.66–1.64)	0.73 (0.46–1.17)	0.73 (0.37–1.47)
**Linezolid**	<0.001		
**CCI ≥1**	1.90 (1.78–2.02)	1.61 (1.51–1.72)	1.61 (1.46–1.78)
**Bronchiectasis**	3.33 (2.85–3.90)	2.30 (1.95–2.71)	2.30 (1.80–2.94)
**History of *H. pylori* infection**	3.76 (2.77–5.10)	1.88 (1.36–2.58)	1.88 (1.17–3.01)

[a] The 99.64% CI reflected that the set type I error was 0.05 divided by 14 given that 14 antibiotics might potentially be used in one patient. Abbreviations: OR, odds ratio; CI: confidence intervals; CCI, Charlson comorbidity index; *H. pylori*, *Helicobacter pylori*.
